# Georgia Medical Association

**Published:** 1872-04

**Authors:** 


### GEORGIA MEDICAL ASSOCIATION.

Atlanta, Georgia, April 26, 1872.

The following extracts from the minutes of the Georgia Medical Association are published by direction of that body, at its last annual meeting, held in Columbus, Georgia, April 10th, 11th and 12th, 1872.

"Dr. Robert Battey offered the following resolution, which was adopted:

"*Resolved*, That the second resolution offered by Dr. Logan be appended to the official letter of Dr. Boring, of the 10th of April, 1869, and published in the two medical journals and one newspaper at Atlanta."

(The letter of Dr. Boring here follows.)

"Atlanta, Georgia, April 10, 1869.

"At a meeting of the Faculty of the Atlanta Medical College of 1868, held in the city of Atlanta, March 16, 1869, the following resolution was unanimously adopted, viz:

"*Resolved*, That this Faculty disavow any purpose to reflect upon the Medical Association of Georgia, either in the 'Memorial' presented to the Legislature of 1868 or upon any other occasion; and that our representatives, who may attend the meeting of said Association to be held in the city of Savannah, on the 14th instant, be, and the same are hereby, instructed to present this disavowal, together with that contained in the 'Memorial.'

"By order of the Faculty. Jesse Boring, M.D., Dean.

"W. S. Armstrong, M.D., Secretary."

(The following is the second resolution introduced by Dr. J. P. Logan, adopted, and referred to in Dr. Robert Battey's resolution:)

"*Resolved*, That we do recognize the regular meeting of the Georgia Medical Association of 1868, in Augusta, as a regular meeting of that body."

J. T. Johnson, M.D.,
*Assistant Secretary Georgia Medical Association.*

